# Reducing Children’s Exposure to Passive Smoking in Rural Communities of Bangladesh: An Application of the Theory of Planned Behavior

**Published:** 2019-12-11

**Authors:** Rishad Choudhury Robin, Narongsak Noosorn

**Affiliations:** ^1^Doctoral Candidate, Faculty of Public Health, Naresuan University, Phitsanulok-65000, Thailand; ^2^Dean and Associate Professor, Faculty of Public Health, Naresuan University, Phitsanulok-65000, Thailand

**Keywords:** Passive smoking, Children, Bangladesh

## Abstract

**Background:** Passive smoking prevalence is high in Bangladesh. We aimed to explore the association of the theory of planned behavior constructs to reduce the exposure of passive smoking among children in the rural area of Bangladesh.

**Study Design:** Cross-sectional study.

**Methods:** Overall, 410 adults had been taken at random following a self-administrative questionnaire. Data had been collected from six villages of Munshiganj district of Bangladesh from Jul to Oct 2018. Descriptive statistics were applied to describe socio-demographic characteristics. Inter correlations coefficient was done to observe the correlation, differences between demographic and dependent variables were assessed for significance using the Chi-square or Fisher’s exact test as appropriate. Univariate and multivariate logistic regression models were used to investigate the association between the theory of planned behavior constructs and exposure of passive smoking among both smoker and nonsmoker groups. All results were presented as unadjusted and adjusted odds ratio (OR) with 95% confidence intervals (CIs). A P-value ≤0.05 was considered as statistically significant.

**Results:** Attitude (OR 0.681, 95% CI: 0.498, 0.931) among smoker and intention (OR 0.226, 95% CI: 0.081, 0.633) was found statistically significant constructs (*P*<0.001) among non-smoker to reduce the exposure. Additionally, the prevalence of passive smoking exposure was found 36.6% (95% CI: 0.32%, 0.41%) on average 40% among males and 28.33% among females.

**Conclusion:** The theory of planned behavior constructs is useful to reduce the exposure of passive smoking among children, which may be useful in the future to design interventions of controlling passives smoking exposure.

## Introduction

Globally, around 1.1 billion people smoke different forms of tobacco smoking products, considered the leading cause of preventable death around the world^1,2^. Worldwide, approximately 7 million people die from direct use of tobacco; however, smoking tobacco is an enormous burden for low and middle-income countries as almost 80% of the world’s smokers living there^2^. Western Pacific region has the highest prevalence of male smokers (48.5%) whereas Europe has the highest incidence of female smokers (19.3%) according to WHO ^3^.

Like smoking, exposure to passive smoking (EPS) is also a public health concern as it is responsible for nearly 1.2 million deaths worldwide^4^. Passive smoking can be defined as inhaling tobacco smoke of other people’s regardless of being a smoker or nonsmoker^5^. China is top in tobacco smoking exposure, where almost 717 million people exposed to passive smoking at home. Next is India (313 million) followed by Indonesia (133 million)^6^. However, children are the most susceptible population to EPS as almost half of the world children are exposed to it^7^. Moreover, the health effect of EPS to children is consistent and robust. EPS is the cause of asthma attack, wheezing, coughing, ear infection, meningococcal disease, lung and cervical cancer, lower respiratory infections in infancy, food allergy, allergic rhinitis, and dermatitis as well as sudden infant death syndrome ^2,8^.

Theory-based health behavior interventions are more effective rather than non-theory based^9,10^. Theory of planned behavior (TPB) is a well-established model to predict an individual’s behavior at a specific place and time^11^. TPB consists of four significant constructs. The intention is the first construct which acts as a motivational factor to follow the particular behavior. Secondly, an attitude reflected a person’s favorable or unfavorable judgment of a given action. Thirdly, subjective norm imitates the social pressure to perform or not to perform a given behavior and finally perceived behavioral control (PBC) which plays a crucial role to determine an individual’s perception of the ease or difficulty of performing the behavior^12^.

Several studies applied TPB as a useful model to predict and explain a wide range of health-related behavior including smoking, drinking, and substance abuse, condom use and others^11^. Additionally, TPB is considered one of the most influential theories applied to understand the psychosocial determinants such as peer influence, cognitive susceptibility, household smoking behavior and attitude of smoking behavior for conducting different kinds of research on smoking as well as designing intervention as physiological determinants of behavior could foster unhealthy behavior^13-15^.

In Bangladesh, the consumption of tobacco products reduced significantly within the last decades though the prevalence of different tobacco products is still 35.3% ^16,17^. Among the total consumers of tobacco, 18% smoke tobacco products while EPS rate among adult is 39% and in between age 13 to15, it is 31.1%^17,18^.

There are few studies regarding EPS among children in Bangladesh and to our knowledge, none of them used TPB to predict the reduction of EPS among children. Thus the theoretical aspect of reducing EPS is still unknown in the context of rural communities of Bangladesh. To replenish the knowledge gap, we aimed to apply the constructs of TPB to predict the behaviors that will help to reduce EPS among children in households located in rural communities of Bangladesh.

## Methods

### Study Design and Sampling

**Figure 1:**
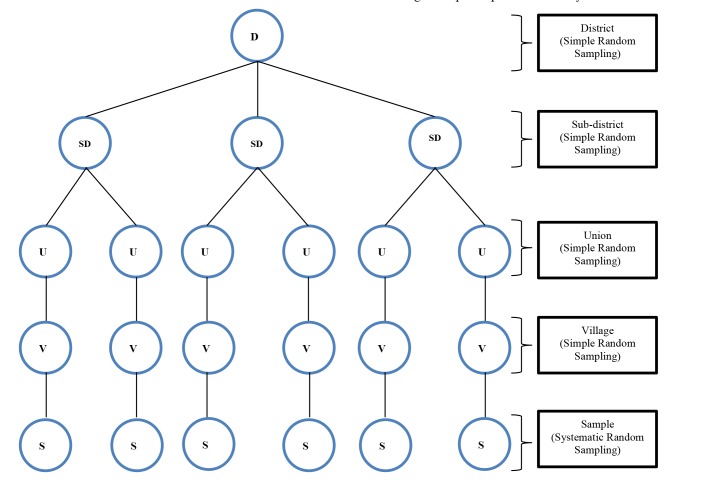
Sampling technique

A cross-sectional survey was carried out in Munshiganj district, Bangladesh from Jul to Oct 2018. Lottery method of simple random sampling was implemented to select Munshiganj district and six villages within the district. Besides, systematic random sampling was applied to select participants from every third household from the selected villages (Figure 1). Districts of hill tracts were not included for the lottery as the majority of the populations of those areas belong to the different socio-cultural, politico-economic and ethnic background from the rest of the country^19^. By using Cochran’s formula, 410 both smokers and non-smokers adult males and females participated in the survey^20^. The inclusion criteria were (i) smoker and non-smoker both male and female who were more than 18 yr old, (ii) those household have at least one child and (iii) resident of the research area staying more than 5 yr continuously and exclusion criteria were (i) those who did not understand the nature of the study and (ii) who disagree to participate in the study.

The study was approved by the Institutional Review Board of Naresuan University, Thailand (IRB Certificate No.0502/61), and all subjects provided informed consent before participation.

### Data Collection

A self-administrative close-ended questionnaire containing several sections including demographics, smoking status, and self-reported exposure in the household, as well as four constructs from TPB which are the intention, attitude, subjective norm, and PBC were used. Demographic variables consist of age, gender, marital status, education, occupation, income, number of children in the household, and tobacco using status of participant and the member of the household. Exposure of passive smoking indicated exposure to another person’s tobacco smoke in the household for at least 15 min daily for more than one day every week in the past 30 days^21^. This study assessed exposure to children from the adult‘s self-report^22,23^.

The TPB constructs include 26 items and three points Likert scale was applied for measuring the data^24^. The TPB constructs were developed according to the directions recommended by Ajzen^25^. The score was calculated three, two, one represented agree, neither agree nor disagree and disagree respectively for positive answers and for negative answers agree, neither agree nor disagree and disagree were represented by one, two, three respectively. The dependent variable was calculated by the score one and zero for exposing to passive smoking and non-expose to passive smoking, respectively.

Regarding the quality of the research tool, content validity by item objective congruence (IOC) and Cronbach’s alpha coefficient were calculated for validity and reliability. For content validity, three experts from tobacco filed were selected. Both IOC and the overall Cronbach’s alpha of TPB constructs were calculated as 0.88 and 0.78, respectively which qualified the acceptable criteria^26,27^. The questionnaire was translated into the native language (Bengali) for easy understanding of the respondents and on average 30 min took by the respondents to complete the survey. The questionnaire was distributed and collected by the principal researcher.

### Statistical Analysis

The data were analyzed using SPSS ver. 20 for Windows (IBM Corp., Armonk, NY). Descriptive statistics were used to describe basic socio-demographic characteristics, intercorrelations coefficient was done to find out the correlation. Differences between demographic variables with the dependent variable were assessed for significance using the chi-square or Fisher’s exact test as appropriate. Univariate and multivariate logistic regression models (Enter method) were used to investigate the association between TPB constructs and EPS among both smoker and nonsmoker groups where demographic variables were included as confounder. TPB constructs were included as continues variable. All results were presented as unadjusted and adjusted odds ratio (OR) with 95% confidence intervals (CIs). A *P*-value ≤0.05 was considered to be statistically significant.

## Results

Our survey on 410 respondents revealed that male participation in the survey was almost two times higher than the female participation, and the prevalence of EPS was 40% and 28.33% for male and female participants, respectively. The prevalence of smoking was found 36.89% among males and 1.6% among females. Respondents age ranged from 18 to 80 yr, and the mean age was 40.39 ± 11.44 (95% CI: 39.28, 41.50) yaers. The most exposed age group was 25-44 yr old (16.09%). Married (32.19%) and those who possess the primary/secondary level of education (26.34%) were more likely to expose. Additionally, people engaged with agriculture sector showed higher exposure (12.68%) to EPS compare to other occupations. Similarly, people with income less than 20,000 BDT were found more prone to EPS. Gender (*P*<0.001), education (*P*<0.001), employment (*P*<0.001) and household income (*P*<0.001) showed a significant difference with EPS reducing (Table 1).

**Table 1:** Socio-demographic and socio-economic characteristics of the sample according to exposure of passive smoking (n=410)

**Variables**	**Total**		**Expose, n=150**		**Non-expose, n=260**		***P-*** **value**
	**Number**	**Percent**	**Number**	**Percent**	**Number**	**Percent**	
**Gender**							0.001
Male	290	70.7	116	28.3	174	42.4	
Female	120	29.3	34	8.3	86	20.9	
**Age ( yr )**							0.051
18-24	38	9.3	17	4.2	21	5.12	
25-44	205	50.0	66	16.1	139	33.9	
45-64	157	38.3	60	14.6	97	23.7	
65+	10	2.4	7	1.7	3	0.7	
**Marital status**							0.679
Single	55	13.4	17	4.1	38	9.3	
Married	351	85.7	132	32.2	219	53.5	
Divorce	1	0.2	0	0.0	1	0.2	
Widow	3	0.7	1	0.2	2	0.5	
**Education**							0.001
No formal schooling	21	5.1	16	3.9	5	1.2	
Primary/ Secondary schooling	235	57.3	108	26.3	127	30.9	
College/ University	154	37.6	26	6.3	128	31.2	
**Employment**							0.001
Service holder	141	34.4	35	8.5	106	25.9	
Business	73	17.8	26	6.4	47	11.5	
Agriculture	84	20.5	52	12.7	32	7.8	
Unemployed	112	27.3	37	9.02	75	18.3	
**Household income in BDT**							0.001
<10000	124	30.0	52	12.7	72	17.6	
10000-20000	186	45.5	78	19.0	108	26.3	
>20000	100	24.5	20	4.9	80	19.5	
**Number of children**							0.736
1	123	30.0	44	10.8	79	19.3	
2-3	241	58.8	89	21.7	152	37.1	
4-5	43	10.5	15	3.7	28	6.8	
6+	3	0.7	2	0.5	1	0.2	

According to table 2, the responded showed relatively higher agreement towards the behavior level of the TPB constructs. Under the TPB constructs, items of intention showed positivity for reducing EPS, but surprisingly, 68.8% of respondents showed the negative intention to a specific topic concerning smoking inside the house. The items of attitude and PBC were positively agreed upon by the respondents to follow the EPS for reducing the behavior. Similarly, items of the subjective norm were also positively agreed by the responded for reducing EPS; except one item (shopkeeper who sells cigarettes will help to reduce exposure) was strongly disagreed by 52% respondents. Total mean scores of TPB constructs found on intention (13.16±1.10), attitude (17.06±1.63), subjective norm (28.38±3.81) and PBC (9.73±1.85) indicated relatively positive behavior to reduce EPS.

**Table 2:** Theory of Planned Behavior constructs

**No**	**Theory of Planned Behavior Constructs**	**Behavior Level (n=410)**						**Mean**	**SD**	**Median** **(IQR)**
		**Agree**		**Neither agree** **nor disagree**		**Disagree**				
		**Number**	**Percent**	**Number**	**Percent**	**Number**	**Percent**			
**A**	**Intention**									
1	No smoking inside house	398	97.1	5	1.7	7	1.2	2.96	0.25	3 (0)
2	No smoking in front of the children	391	95.4	12	2.9	7	1.7	2.94	0.31	3 (0)
3	Spouse will not smoke while children present	397	96.8	10	2.4	3	0.8	2.96	0.23	3 (0)
4	Banning smoking inside house	361	88.0	28	6.8	21	5.2	2.83	0.49	3 (0)
5	Specify a place for smoking in the house	67	16.3	61	14.9	282	68.8	1.48	0.76	1 (1)
**B**	**Attitude**									
1	People will not smoke inside the house	370	90.2	20	4.9	20	4.9	2.85	0.47	3 (0)
2	People will not smoke in front of children	387	94.4	18	4.4	5	1.2	2.93	0.30	3 (0)
3	Not a big deal to smoke in front of a children	311	75.9	62	15.1	37	9.0	2.67	0.64	3 (0)
4	Children should never be exposed to smoke	392	95.6	8	2.0	10	2.4	2.93	0.34	3 (0)
5	Parents responsibility is to teach a child about smoking exposure	388	94.6	13	3.2	9	2.2	2.92	0.34	3 (0)
6	Confidence of the ability to become (or stay) nonsmokers	341	83.2	36	8.8	33	8.0	2.75	0.59	3 (0)
**C**	**Subjective Norm**									
1	Spouse will help to reduce the exposure	358	87.3	38	9.3	14	3.4	2.84	0.45	3 (0)
2	Parents and in-laws will help to reduce the exposure	333	81.3	58	14.1	19	4.6	2.77	0.52	3 (0)
3	Other family members will help to reduce the exposure	343	83.7	46	11.2	21	5.1	2.79	0.52	3 (0)
4	Friends will help to reduce the exposure	287	70.0	70	17.1	53	12.9	2.57	0.71	3 (1)
5	Children will help to reduce the exposure	363	88.5	32	7.8	15	3.7	2.85	0.45	3 (0)
6	Community health workers will help to reduce the exposure	313	76.3	51	12.5	46	11.2	2.65	0.67	3 (0)
7	Religious leader will help to reduce the exposure	311	75.9	62	15.1	37	9.0	2.67	0.64	3 (0)
8	Political leader will help to reduce the exposure	202	49.2	104	25.4	104	25.4	2.24	0.83	2 (2)
9	Teacher will help to reduce the exposure	375	91.5	22	5.3	13	3.2	2.88	0.41	3 (0)
10	Shopkeeper who sells cigarette will help to reduce the exposure	109	26.6	88	21.4	213	52.0	1.75	0.85	1 (2)
11	Co-worker will help to reduce the exposure	237	57.8	95	23.2	78	19.0	2.39	0.79	3 (1)
**D**	**Perceived Behavioral Control**									
1	Confident to reduce exposure inside the house	367	89.5	29	7.1	14	3.4	2.86	0.43	3 (0)
2	No control to reduce the exposure	255	62.2	78	19.0	77	18.8	2.43	0.79	3 (1)
3	Motivate others to control smoking exposure inside the house	204	49.8	135	32.9	71	17.3	2.32	0.75	2 (1)
4	Impossible to motivate others to control smoking exposure inside the house	170	41.5	117	28.5	123	30.0	2.11	0.75	2 (2)

TPB depicted the factors related to the reduction of EPS. The relationship between TPB constructs and reducing EPS ascertained that reducing EPS showed to be moderately negatively correlated with attitude (0.35) and PBC (0.27). The EPS is mostly influenced by people attitude and PBC (Table 3).

**Table 3:** Inter correlation coefficient between the theory of planned behavior contracts and reducing exposure of passive smoking

**Variable**	**Intention**	**Attitude**	**Subjective Norm**	**Perceived Behavioral Control**	**Reducing Exposure** **of Passive Smoking**	**Mean**	**SD**
Intention	1.000					13.16	1.10
Attitude	0.306	1.000				17.06	1.63
Subjective Norm	0.288	0.273	1.000			28.38	3.81
Perceived Behavioral Control	0.086	0.324	0.338	1.000		9.73	1.85
Reducing Exposure of Passive Smoking	-0.120	-0.350	-0.196	-0.279	1.000	0.37	0.48

When analyzed with logistic regression, attitude (OR 0.681, 95% CI: 0.498, 0.931) among smoker was found statistically significant predictors (*P*<0.001) for reducing EPS. The overall model prediction showed that 81.3% of the cases were correctly classified into the observed categorized of the dependent variable. The Hosmer and Lemeshow test were showed as 0.368 and Nagelkerke R Square was 0.191 (Table 4).

**Table 4:** Logistic regression: Odds ratios (ORs) and 95% confidence intervals (CI) for the constructs of the theory of planned behavior related to reduce passive smoking exposure among smokers (n=171)

**Variables**	**Unadjusted OR (95%CI)**	***P *** **value**	**Adjusted OR (95%CI)** ^a^	***P*** **value**
Intention	0.974 (0.742, 1.278)	0.849	1.131 (0.746, 1.714)	0.561
Attitude	0.711 (0.537, 0.940)	0.001	0.681 (0.498, 0.931)	0.001
Subjective N orm	1.008 (0.921, 1.103)	0.856	1.064 (0.946, 1.198)	0.302
Perceived Behavioral Control	0.981 (0.821, 1.171)	0.829	0.979 (0.789, 1.214)	0.846

^a^ Adjusted for age, sex, marital status, education, occupation, income and number of children

Similarly, intention (OR 0.226, 95% CI: 0.081, 0.633) was found statistically significant predictors (*P*<0.001) among non-smoker for reducing EPS. The overall model prediction was 94.5% as well the Hosmer and Lemeshow test, and Nagelkerke R Square were showed as 0.780 and 0.368 respectively (Table 5).

**Table 5:** Logistic regression: Odds ratios (ORs) and 95% confidence intervals (CI) for the constructs of the theory of planned behavior related to reduce passive smoking exposure among non-smokers (n=239)

**Variables**	**Unadjusted OR (95%CI)**	***P *** **value**	**Adjusted OR (95%CI)** ^a^	***P*** **value**
Intention	0.313 (0.161, 0.607)	0.001	0.226 (0.081, 0.633)	0.001
Attitude	0.725 (0.429, 1.226)	0.230	0.866 (0.399, 1.877)	0.715
Subjective Norm	0.852 (0.739, 0.982)	0.027	0.842 (0.690, 1.028)	0.091
Perceived Behavioral Control	0.835 (0.565, 1.234)	0.365	0.853 (0.514, 1.416)	0.539

^a^ Adjusted for age, sex, marital status, education, occupation, income and number of children

## Discussion

TPB is considered as a useful model to predict human behavior and widely used in smoking, alcohol, substance abuse as well as other areas of health^9,28,29^. Our study findings reflected that attitude and intention were significant constructs of TPB to reduce the EPS among children. The finding is similar to the studies conducted in Iran showed attitude was a potential factor to predict smoking and drug abuse-related behavior among adolescent^30-32^. Furthermore, other studies conducted to predict the condom using behavior, in the USA on college student reflected attitude and intention imitated the significant^33^. In Israel, a study conducted among pregnant women also consistent with our finding indicated that attitude was significant predictors to predict the intention of smoking^34^. In the same line to our finding, attitude also considered as significant predictors for preoperative smoking abstinence^35^. Furthermore, another study among high-risk lung cancer patient, the intention was favorable to reduce passive smoking exposure^36^. Moreover, intention was significant to quit smoking among mentally ill young adult smokers^37^ as well as intention could be predicted to smoke cigarettes among water pipe smoker^38^. In Iran, intention was influential predictors on doing regular pap-smear test^39^. Therefore, our results explained that the TPB model is useful to reduce EPS.

In addition, our study found that the overall prevalence of EPS was 36.6% in the household, comparable to the results from global adult tobacco survey (GATS), which showed a prevalence of 39.0%^17^. The prevalence is lower than neighboring countries India and Myanmar indicating 38.7% and 39.1% respectively^40,41^. Prevalence of smoking of tobacco was found 25.6%, however; compared to the GATS data indicate 18.0%^17^. A possible explanation for this difference could be that our study’s result imitated only a specific district whereas the GATS data represent the whole country. Our study showed that among the age group 25 to 44 yr, the exposure of passive smoking was high. However, another study conducted among adults of Bangladesh found age group 15 to 24 yr were more likely to expose to passive smoking^42^. The difference of the result may be due to that our study conducted on both smoker and non-smoker group in a specific district whereas the previous study was conducted on only non-smoker adults in six divisions. Our study also found that those who completed 10 or more years of education were less exposed to EPS. The result is also in line with the finding of a study conducted on the young adults of Bangladesh showed that those who completed 9 or more years of education were 6.7 times less likely to expose to passive smoking^43^. Moreover, our study also imitated that people with high income were less exposed to passive smoking. Research conducted among women of Bangladesh also confirmed this finding that lower and middle-income people were more exposed to passive smoking^44^. Our findings also reflected that those who work in the agriculture sector were more prone to EPS. It may due to those who work in the agricultural sector have low educational attainment, which has an increased chance of passive smoking exposure.

The study result faces some limitations. The research is a cross-sectional study, and all independent and dependent variables were measured in a single point of time. All variables were self-reported, which may lead to misclassification due to recall and reporting bias. Additionally, the study was only concentrated on household factors. Other settings, such as restaurants, workplaces, or public places, were not included in the study whereas these are potential places for EPS. Moreover, the survey conducted in a specific district of Bangladesh, which may not be the reflection of whole Bangladesh rather it may reflect most of the plain land districts of the country.

Regardless of these limitations, this study has advantages. The research was conducted in two groups, including smoker and non-smoker. This may reduce the potential bias and the sample was likely to be highly representative of the population that was studied.

## Conclusion

EPS is a problematic fact worldwide as well as Bangladesh and needs to be concerned as children are the most vulnerable population. Cognition is a critical process to practice good health behavior. Theoretical based research can help to understand the cognition elements better. The TPB constructs of intention and attitude explain significant to reduce EPS among children. Since smoking is a common practice in Bangladesh, TPB could be used as a key model to reduce the exposure of passive smoking in the household thus helps the child live a better and healthy life. This information may be useful in the future design of interventions of tobacco control in the aspect of passives smoking exposure.

## Acknowledgements

The first author acknowledges Naresuan University, Thailand to award him Naresuan University International Student Scholarship 2016 for pursuing Doctor of Public Health degree. Both authors also acknowledge the anonymous reviewers for their critical and constructive comments for the development of the manuscript.

## Conflict of interest

There is no Conflict of interest by the authors.

## Funding

There is no funding from any organization.

## Highlights

- High prevalence of Exposure to Passive Smoking (EPS) in the rural areas of Bangladesh.
- Attitude and intention were significant predictors to reduce EPS.
- TPB is a useful framework to reduce EPS among children.

## References

[1] World Health Organization. Global Health Observatory (GHO) data. WHO Web Site; 2019 [updated 2019; cited 17 July 2019]; Available from: https://www.who.int/gho/tobacco/use/en/.

[2] Centers for Disease Control and Prevention. Smoking & Tobacco Use, Health Effects of Secondhand Smoke. CDC Web Site; 2018 [updated 17 Jan 2018; cited 5 Feb 2019]; Available from: https://www.cdc.gov/tobacco/data_statistics/fact_sheets/secondhand_smoke/health_effects/index.htm.

[3] World Health Organization. Global Health Observatory data repository. WHO Web Site; 2019 [updated 2016; cited 17 July 2019]; Available from:http://apps.who.int/gho/data/view.main.1805REG?lang=en.

[4] World Health Organization. Tobacco. WHO Web Site; 2019 [updated 26 July 2019; cited 3 Aug 2019]; Available from: https://www.who.int/news-room/fact-sheets/detail/tobacco.

[5] Cancer Research UK. What is passive smoking? Cancer Research UK Web Site; 2019 [updated 28 Dec 2018; cited 17 July, 2019]; Available from: https://www.cancerresearchuk.org/about-cancer/causes-of-cancer/smoking-and-cancer/what-is-passive-smoking.

[6] Global Adult Tobacco Survey. Exposure to Smoke: Home. GATS Web Site; 2019 [updated 2015; cited 18 July, 2019]; Available from: http://gatsatlas.org/pdf/mobile/index.html#p=55.

[7] World Health Organization. Tobacco free initiative consultation report. WHO Web Site; 1999 [updated 1999; cited 6 March 2019]; Available from: https://apps.who.int/iris/bitstream/handle/10665/65930/WHO_NCD_TFI_99.10.pdf?sequence=1&isAllowed=y.

[8] Cao S, Yang C, Gan Y, Lu Z (2015). The Health Effects of Passive Smoking: An Overview of Systematic Reviews Based on Observational Epidemiological Evidence. PLoS One.

[9] Webb TL, Sniehotta FF, Michie S (2010). Using theories of behaviour change to inform interventions for addictive behaviours. Addiction (Abingdon, England).

[10] Glanz K, Bishop DB (2010). The role of behavioral science theory in development and implementation of public health interventions. Annu Rev Public Health.

[11] Boston University School of Public Health. The theory of planned behavior. Boston University Web Site; 2018 [updated 9 Sept 2019; cited 7 February 2019]; Available from: http://sphweb.bumc.bu.edu/otlt/MPH-Modules/SB/BehavioralChangeTheories/BehavioralChangeTheories3.html.

[12] Ajzen I (1991). The theory of planned behavior. Organ Behav Hum Decis Process.

[13] Topa G, Moriano JA (2010). Theory of planned behavior and smoking: meta-analysis and SEM model. Subst Abuse Rehabil.

[14] Macleod J, Davey SG (2003). Psychosocial factors and public health: a suitable case for treatment?. J Epidemiol Community Health.

[15] Talluri R, Wilkinson AV, Spitz MR, Shete S (2014). A risk prediction model for smoking experimentation in Mexican American youth. Cancer Epidemiology Biomarkers and Prevention.

[16] World Health Organization. Global adult tobacco survey comparison fact sheet Bangladesh 2009 and 2017. WHO Web Site; 2018 [updated 7 Aug 2018; cited 6 Feb 2019]; Available from: http://bbs.portal.gov.bd/sites/default/files/files/bbs.portal.gov.bd/page/57def76a_aa3c_46e3_9f80_53732eb94a83/GATS_BAN_2017_Comparison_Fact%20Sheet.pdf.

[17] World Health Organization. Global adult tobacco survey fact sheet Bangladesh 2017. WHO Web Site; 2018 [updated 7 Aug 2018; cited 7 Feb 2019]; Available from: https://www.who.int/tobacco/surveillance/survey/gats/fact-sheet-gats-bangladesh-2017.pdf?ua=1.

[18] World Health Organization. Global youth tobacco survey Bangladesh report 2013. WHO Web Site; 2015 [updated 2015; cited 7 Feb 2019]; Available from: https://apps.who.int/iris/bitstream/handle/10665/164335/9789290224815-GYTS-TFI.pdf?sequence=1&isAllowed=y.

[19] Nasir U (2012). Politics of Cultural Difference: Identity and Marginality in the Chittagong Hill tracts of Bangladesh. South Asian Survey.

[20] Cochran WG. Sampling Techniques. 3d ed. New York: John Wiley & Sons; 1977.

[21] Yang L, Tong EK, Mao Z, Hu TW (2010). Exposure to secondhand smoke and associated factors among non-smoking pregnant women with smoking husbands in Sichuan province, China. Acta Obstet Gynecol Scand.

[22] Zafar Ullah AN, Huque R, Akter S, Nasreen S, Akter H, Thomson H (2013). Children's exposure to second-hand smoke at home in Bangladesh: a community survey. BMJ Open.

[23] Avila-Tang E, Elf JL, Cummings KM, Fong GT, Hovell MF, Klein JD (2013). Assessing secondhand smoke exposure with reported measures. Tob Control.

[24] Srithongthum J. Factor affecting exercise behavior of people exercising at Lumpini Park, Bangkok [Master’s thesis]. Bangkok: Srinakharinwirot University; 2007. [in Thai].

[25] Ajzen I. Constructing a TPB questionnaire: conceptual and methodological considerations 2002; Available from: https://pdfs.semanticscholar.org/0574/b20bd58130dd5a961f1a2db10fd1fcbae95d.pdf?_ga=2.138591475.1739291134.1575200904-1445690292.1575200904.

[26] Turner R, Carlson L (2003). Indexes of item-objective congruence for Multidimensional Items. Int J Test.

[27] Chakkraphan P, Boonchanuttha P, Jariya H, Warangaungkana M (2018). Predicting factors for smoking behavior among women who frequent nightlife entertainment venues around a university in the northern region of Thailand. Subst Abuse.

[28] Murnaghan DA, Blanchard CM, Rodgers WM, LaRosa JN, MacQuarrie CR, MacLellan DL (2010). Predictors of physical activity, healthy eating and being smoke-free in teens: A theory of planned behaviour approach. Psychol Health.

[29] Cooke R, Dahdah M, Norman P, French DP (2016). How well does the theory of planned behaviour predict alcohol consumption? A systematic review and meta-analysis. Health Psychol Rev.

[30] Bashirian S, Hidarnia A, Allahverdipour H, Hajizadeh E (2012). Application of the theory of planned behavior to predict drug abuse related behaviors among adolescents. J Res Health Sci.

[31] Karimy M, Niknami S, Heidarnia AR, Hajizadeh I, Montazeri A (2013). Prevalence and Determinants of Male Adolescents’ Smoking in Iran: An Explanation Based on the Theory of Planned Behavior. Iran Red Crescent Med J.

[32] Karimy M, Niknami S, Hidarnia AR, Hajizadeh I (2012). Intention to start cigarette smoking among Iranian male adolescents: usefulness of an extended version of the theory of planned behavior. Heart Asia.

[33] Asare M (2015). Using the theory of planned behavior to determine the condom use behavior among college students. Am J Health Stud.

[34] Ben NM, Golubev V, Shamrai V (2010). Smoking during pregnancy: analysis of influencing factors using the theory of planned behaviour. Int Nurs Rev.

[35] Shi Y, Ehlers S, Warner DO (2014). The theory of planned behavior as applied to preoperative smoking abstinence. PLoS One.

[36] Manning M, Wojda M, Hamel L, Salkowski A, Schwartz AG, Harper FW (2017). Understanding the role of family dynamics, perceived norms, and lung cancer worry in predicting second-hand smoke avoidance among high-risk lung cancer families. J Health Psychol.

[37] Brunette MF, Ferron JC, Aschbrenner KA, Pratt SI, Geiger P, Kosydar S (2019). Attitudes about smoking cessation treatment, intention to quit, and cessation treatment utilization among young adult smokers with severe mental illnesses. Addict Behav.

[38] Alanazi NH, Lee JW, Dos Santos H, Job JS, Bahjri K (2017). The use of planned behavior theory in predicting cigarette smoking among Waterpipe smokers. Tob Induced Dis.

[39] Jalilian F, Emdadi S (2011). Factors Related to Regular Undergoing Pap-smear Test: Application of Theory of Planned Behavior. J Res Health Sci.

[40] World Health Organization. Global adult tobacco survey fact sheet India 2016-17. WHO Web Site; 2017 [updated 10 Oct 2017; cited Feb 21 2019]; Available from: https://www.who.int/tobacco/surveillance/survey/gats/GATS_India_2016-17_FactSheet.pdf?ua=1.

[41] World Health Organization. Factsheet 2018 Myanmar. WHO Web Site; 2017 [cited 21 Mar 2019]; Available from: https://apps.who.int/iris/bitstream/handle/10665/272686/wntd_2018_myanmar_fs.pdf;jsessionid=4A302D0D699A9E102C4B3B2CD6C56D3C?sequence=2.

[42] Abdullah AS, Driezen P, Sansone G, Nargis N, Hussain GA, Quah AC, Fong GT (2014). Correlates of exposure to secondhand smoke (SHS) at home among non-smoking adults in Bangladesh: findings from the ITC Bangladesh survey. BMC Pulm Med.

[43] Sultana R, Akter J, Nahar N, Faruque M, Begum R, Ahmed M (2016). Perceptions about the health effects of passive smoking among Bangladeshi young adults. Int J Stat Med Res.

[44] Fischer F, Minnwegen M, Kaneider U, Kraemer Kraemer, Khan MM (2015). Prevalence and determinants of secondhand smoke exposure among women in Bangladesh, 2011. Nicotine Tob Res.

